# Tissue remodeling investigation in varicose veins

**Published:** 2012

**Authors:** Sayyed Mohammad Hossein Ghaderian, Zohreh Khodaii

**Affiliations:** 1 *Department of Medical Genetics, Faculty of Medicine, Shahid Beheshti University of Medical Sciences & Health Services,Tehran, Iran*; 2 *Department of Biochemistry, Nutrition, Medical Genetics, Faculty of Medicine, Alborz University of Medical Sciences & Health Services, Karaj, Alborz*

**Keywords:** Varicose vein disease, endothelial cells, transmission electron microscopy, collagen fibres

## Abstract

Although the etiology of varicose veins remains unknown, recent studies have focused on endothelial cell integrity and function because the endothelium regulates vessel tone and synthesizes many pro- and anti-inflammatory factors. The aim of this study was to investigate the evidence involving the endothelium in the development of varicose vein disease. In addition, tissue remodeling was investigated in varicose veins to determine the expression of different types of collagen. Tissue specimens of superficial varicose veins and control saphenous vein were used for immunohistochemical and transmission electron microscope (TEM). α-smooth muscle actin, and collagen I, III, IV antibodies were applied for immunohistochemical investigation. Findings of this study showed alterations of the intima, such as focal intimal discontinuity and denudation of endothelium; and the media, such as irregular arrangements of smooth muscle cells and collagen fibres in varicose veins. Our findings showed some changes in terms of distribution of types I, III and IV collagen in the intima and media of varicose vein walls compared with controls. These alterations to the media suggest that the pathological abnormality in varicose veins may be due to the loss of muscle tone as a result of the breakup of its regular structure by the collagen fibres. These findings only described some changes in terms of distribution of these types of collagen in the intima and media of varicose vein walls which may result in venous wall dysfunction in varicosis.

Varicose vein disease is a disorder of the lower extremities (1) characterised by reflux in the deep veins which results from a decrease in the venous muscle tone (2). Veins become dilated, cusp insufficiency occurs, valve incompetence follows and high venous pressure results (3). The prevalence of varicose veins is higher in females than in males although it has been suggested that the sex ratio decreases with increasing age (4). Histological investigation of the varicose vein wall has demonstrated a disruption of the organisation of the extracellular matrix and smooth muscle architecture, characterised by separation, degeneration and interruption of the muscular bundles. This is accompanied by varying degrees of intimal thickening, infiltration of fibrous tissue in the muscle layers and marked thinning of the vessel wall at the site of the varices (5,6). Changes in the venous wall function and valve incompetence lead to venous stasis, relative hypoxia, endothelial activation, adhesion molecules expression, accumulation of connective tissue and increased matrix protein expression, and proliferation of smooth muscle cells in the media of the varicose veins (7). These synthetic smooth muscle cells synthesize larger amounts of the extracellular matrix components and lose the expression of the contractile filaments leading to the thickening of the venous wall and loss of the contractility in the varicose vein (8). However, others believe there is a reduction in the cellularity of the smooth muscle layer with replacement by collagen or a significant increase in collagen content of varicose veins (9,10). In normal veins, the extracellular matrix of medial layer, including collagen and elastic fibres, is produced primarily by smooth muscle cells and in adventitial layer, collagen fibres are synthesized and secreted by the adventitial fibroblasts (11).

Although endothelial cells are able to synthesize basement membrane and interstitial collagen, the principal source of collagen in the vessel wall is the smooth muscle cell. Alteration of smooth muscle cells behaviour from quiescent or “contractile state” typical of the normal vessel phenotype to a proliferative or “synthetic state” characteristic of the atherosclerotic phenotype increases collagen synthesis (12).

It was found that in this situation, type I collagen synthesis increases. Similar collagen changes occur in phenotypically altered smooth muscle cells in hypertension (13). The amount of collagen and elastin in the veins, and especially the saphenous veins, contain more collagen than elastin (47% and 7% of the dry weight for collagen and elastin, respectively) (10). The predominant vascular collagens are types I and III, which comprise up to 80-90% of the total blood vessel wall collagens (Type I collagen about 60% and type III about 30% of the total collagen (14). Type IV collagen is a major component of the basal lamina of blood vessels, which plays an important role in regulating pro- and anti-angiogenic events (15). It is distributed in sub-endothelial cells of intima and around smooth muscle cells in the media and in the basement membrane of the *vasa vasorum *and nerve fibres in the adventitia (16).

Moreover, there is some variation in the amount and location of vascular collagen in different forms of vascular diseases (17). For instance, larger areas and higher amounts of collagen were identified in varicose veins compared to controls (18). Kirsch et al. have shown that there is significant increase in matrix proteins such as type IV collagen and laminin in the wall of varicose veins compared with normal veins (19). However, some investigations have indicated a deficiency in collagen such as type III collagen in this disease (20). Moreover, the imbalance in the synthesis of type I collagen and type III collagen can affect vein wall function in varicose veins as described in “the weak wall hypothesis”. It has been shown that type I collagen is significantly increased in affected and unaffected segments of varicose veins compared with control saphenous veins (21).

In addition, to investigate tissue remodeling in varicose veins, the expression of different types of collagen was examined. Furthermore, because of the conflicting reports on the changes of structure in varicose veins, this study aimed to characterise alterations of both microscopic and ultramicroscopic morphology of varicose veins compared with control saphenous vein.

## Materials and methods

Saphenous vein specimens were obtained from 20 patients (7F/13M; age range 43-74 years) undergoing long saphenous vein harvesting for coronary artery bypass grafting at the Shahid Modarress Hospital in Tehran, Iran. All patients had some degree of high blood pressure, atherosclerosis and heart disease. Tissue specimens were obtained from 20 patients (11F/9M; age range 31-77 years) with superficial varicose veins, undergoing surgery for varicose veins at the Wharfedale General Hospital at Otley. The diagnosis of primary varicose veins was made by the referring surgeon and confirmed by venous duplex scanning. These were defined as clinically evident varicose veins, CEAP grade 3 or above. Appropriate ethical approval and informed consent was obtained in all cases.

**Immunohistology**

Control and patient saphenous vein was snapped frozen in OCT (Raymond A Lamb, UK.) then 5μm frozen sections were cut using a Cryostat (LEICA CM 1800), placed onto Poly-L-Lysine (0.1%) (Sigma-Aldrich Company Ltd. UK.) coated slides, and used for immunohistochemical analysis. Immunostaining was carried out as described previously (22,23). The primary antibodies, (α-smooth muscle actin (Novocastra Laboratories Ltd. UK.), collagen type I (Abcam Ltd. UK.), III (Abcam Ltd. UK.), IV (Dako Ltd. UK.), were then applied at appropriate concentration and for the appropriate incubation times. The sections were then incubated with a 1:200 dilution of secondary antibody (peroxidase-conjugated goat anti-mouse immunoglobulin; Dako Ltd. UK.) containing 5% normal human serum diluted in PBS, after washing in PBS for 5 minutes.Slides were examined with a Nikon (ECLIPSE 80i) light microscope and photographs were taken with a Nikon DS-5MC digital camera and ACT-2U software.

**Electron Microscopy**

For electron microscopy the tissue was washed with PBS and then cut into blocks no bigger than 1mm3 (the vein was cut into small pieces cross-sectionally). Preparation of sections was carried out as describedpreviously (24,25). The tissue was incubated in uranyl acetate (26) (BDH-VWR International Ltd. UK.) for 15 minutes to improve contrast in the staining of the cell membrane. The blocks were cut out of the capsules and cutinto 70nm sections using an ultra cut microtome (Reichert-Jung; Austria, type: 701701). Then the sections were mounted on 3.50 mm copper grids and stained with Reynolds Lead Citrate, to improve contrast and staining.A Jeol 1200 EX (Japan, 1970) Transmission Electron Microscope was used for examining E.M. specimens.

## Results

**Immunohistology**

**Expression of α -Smooth Muscle Actin**

**Fig 1:**
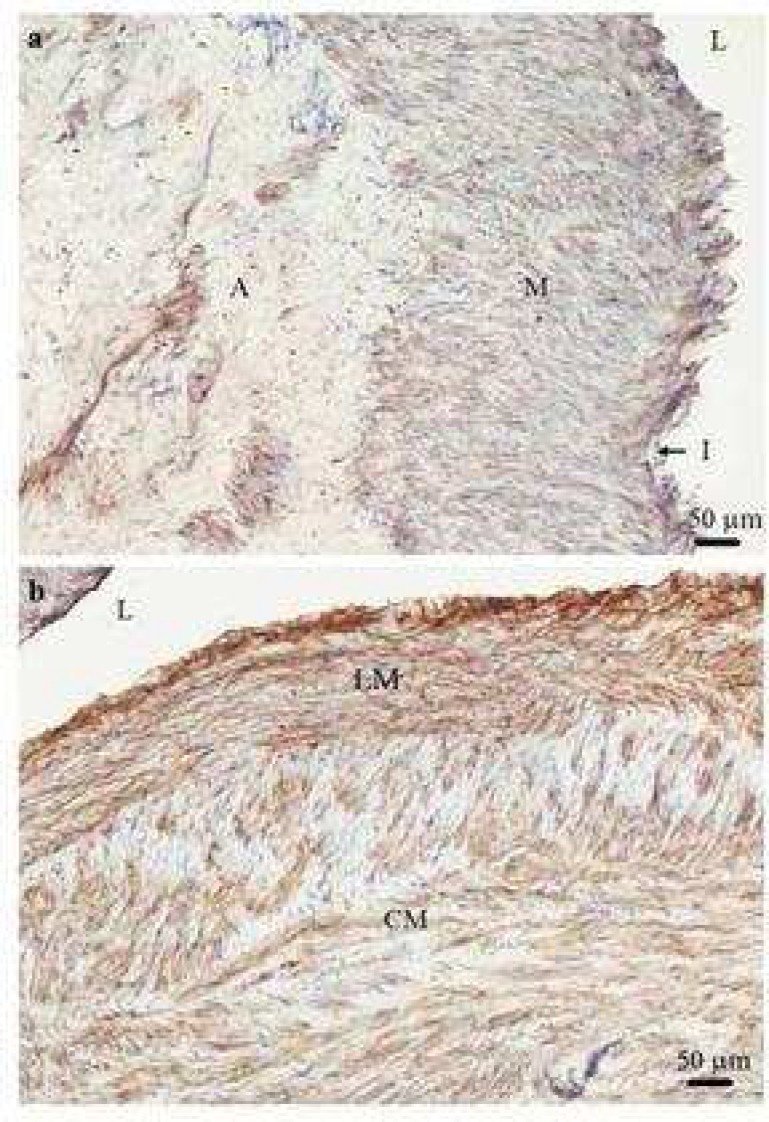
Smooth muscle cells staining with α -smooth muscle actin in varicose vein. A transverse section of varicose vein stained with α -smooth muscle actin antibody showing the media contains no illustrious bundles of longitudinal (LM) and circular smooth muscles (CM) (a). Moreover longitudinal and circular smooth muscle cells cannot be distinguished as complete separate inner and outer bundles within the media (b). I: intima; A: adventitia; L: lumen of the vein; M: muscles (×100).

**Fig 2:**
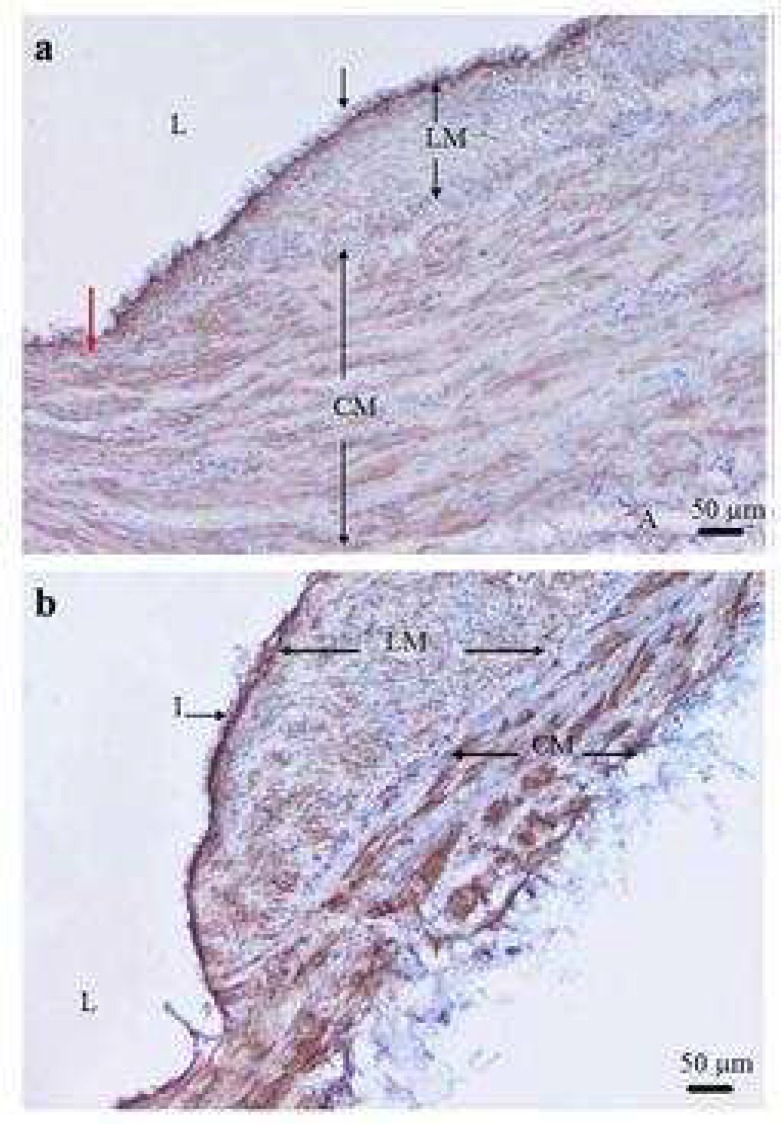
Variation of thickness of longitudinal and circular smooth muscle cells in varicose vein. A transverse section of varicose vein stained (brown) with α -smooth muscle actin antibody showing the longitudinal smooth muscle cell (LM) is thicker on right side than left side (red arrow) of specimen (a) and thickness of two parts of LM bundle is different. The LM layer in some parts of vein wall is thicker than the circular smooth muscle cell (CM) layer (b). I: intima; A: adventitia; L: lumen of the vein (×100

α-SMA antibodies were used to demonstrate the presence of smooth muscle cells. All of the smooth muscle layers in the veins reacted strongly with α -SMA. The individual smooth muscle bundles were completely separated and located close to the intima in all of the control saphenous vein specimens. Longitudinal (LM) and circular (CM) smooth muscle bundles in the media were recognisable in thirteen (65%) varicose vein specimens. The LM and CM bundles were not distinguished in seven (35%) varicose veins specimens as separate bundles when compared to other varicose and control saphenous vein specimens (Fig.1).The inner layer of media varied in thickness in different parts of the vein wall but in 8 out of 20 varicose vein specimens (40%) appeared thicker than the outer layer in some parts of the media (Fig.2a,b). The circular smooth muscle was disorganized and the thickness of its bundles appeared to be reduced in 4 (20%) varicose vein specimens (Table1).

**Table 1:** A summary of the results of staining of varicose veins and control saphenous veins wall for α-smooth muscle actin, types I, III, and IV collagen antibodies

**Type of Antibody**	** Type of Tissue**	
	**Varicose Veins**	**Control saphenous veins**
α-smooth muscle actin	65% organized SMCa bundles	100% organized SMC bundles
Type I collagen	90% IPb subendothelial 45% IP Mc&Ad	100% IP subendothelial 30% IP M&A
Type III collagen	100% IP subendothelial 85% IP M&A	100% IP subendothelial 100% IP M&A
Type IV collagen	100% IP intima 80% α -SMAe staining feature	100% IP intima 100% α-SMA staining feature

a; smooth muscle cell          b; Intensive positive           c; media

d; adventitia           e; α-smooth muscle actin

**Types I, III and IV collage**n

**Fig 3:**
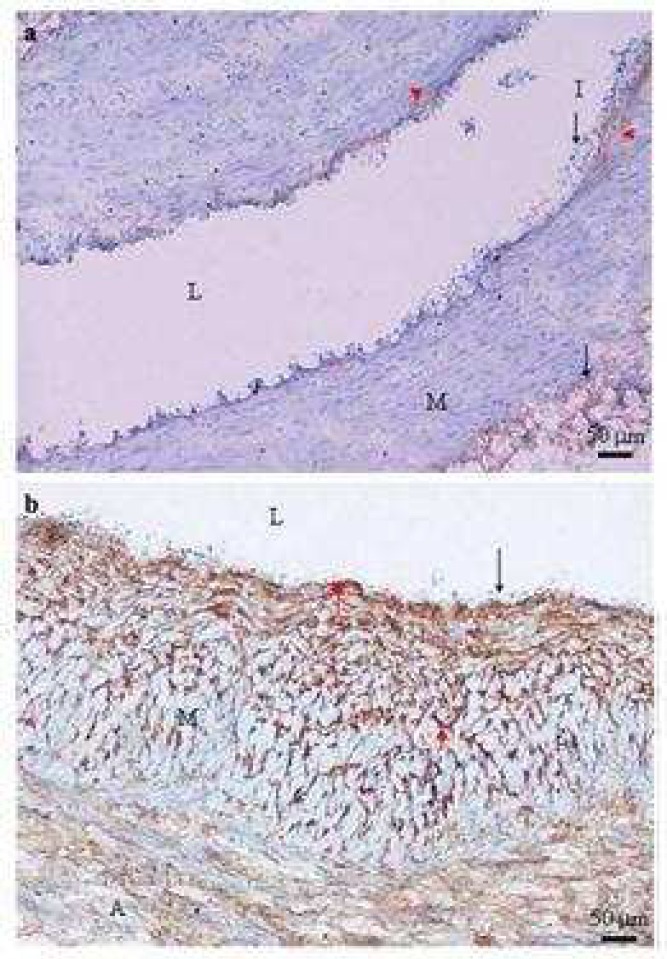
Negative expression of type I collagen in the media of varicose vein (a) and distribution of type I collagen in varicose vein (b). Typical transverse section of varicose veins stained with type I collagen antibody show poor (a) and strong (b) positive staining (brown) which was identified in the subendothelial layer (red arrow heads), adventitia (arrow), and media (M) (a). Type I collagen positive staining was not detected in the media (M) of the varicose vein. I: intima; L: lumen of the vein (×100

**Fig 4:**
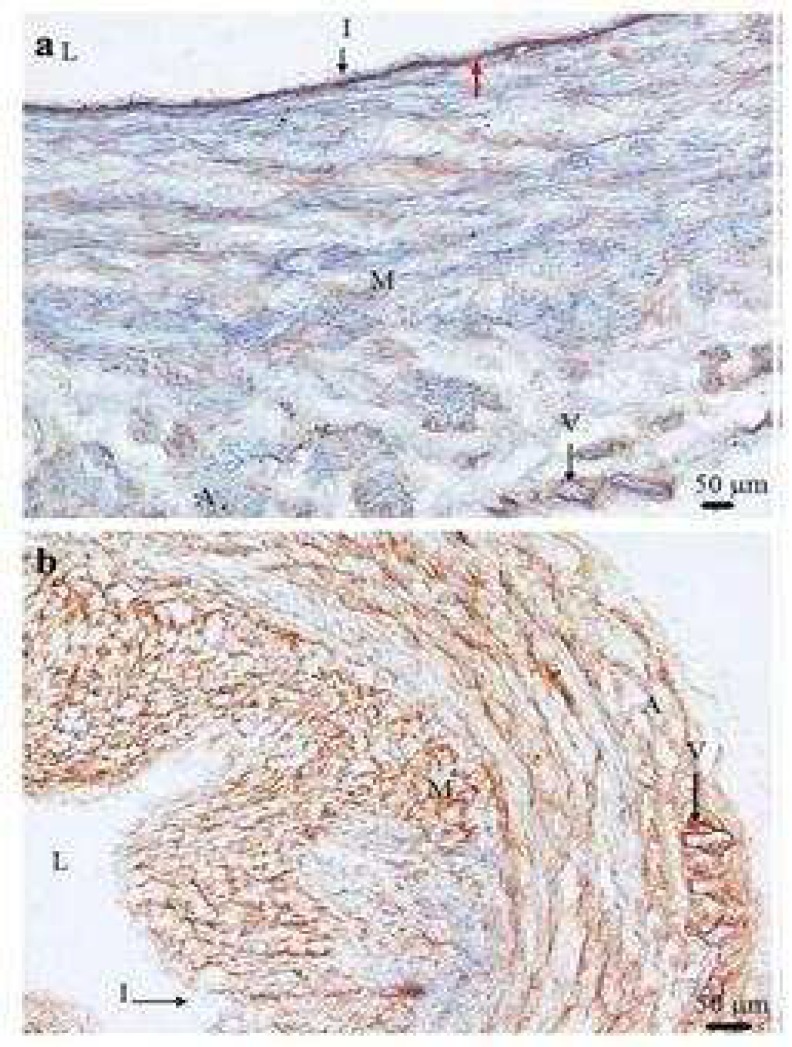
Type I collagen expression in control saphenous vein (a) and distribution of type I collagen in control saphenous vein (b). Typical transverse section of control saphenous vein stained with type I collagen antibody showing strong positive staining (brown) which was detected in subendothelial layer (red arrow) (a) and through the vein wall except the intima (I) (b). It also shows the poor positive staining of type I collagen in the media (M) and the adventitia (A) compared with subendothelial layer staining of type I collagen. In the media (M) and the adventitia (A) layers, intensive staining of type I collagen was shown. I: intima; V: vessel of *vasa vasorum*; L: lumen of the vein (×100

Intensive staining of type I collagen was seen in the subendothelial region of 9 of 20 specimens (45%) of varicose vein. Less intensive staining was seen in the rest of vein wall in the same specimens (Fig.3a). A further 9 specimens of varicose veins (45%) showed uniform staining throughout the vein wall (Fig.3b). Two specimens (10%) of varicose vein showed weak staining of type I collagen in only the subendothelial region and adventitia of vein wall. In 14 specimens (70%) of control saphenous vein, the subendothelial region showed the most intensive staining of type I collagen compared with the rest of vein wall whereas uniform intensive staining was visible in the media and the adventitia of the other 6 specimens (30%) (Fig.4a,b) (Table 1).

**Fig 5:**
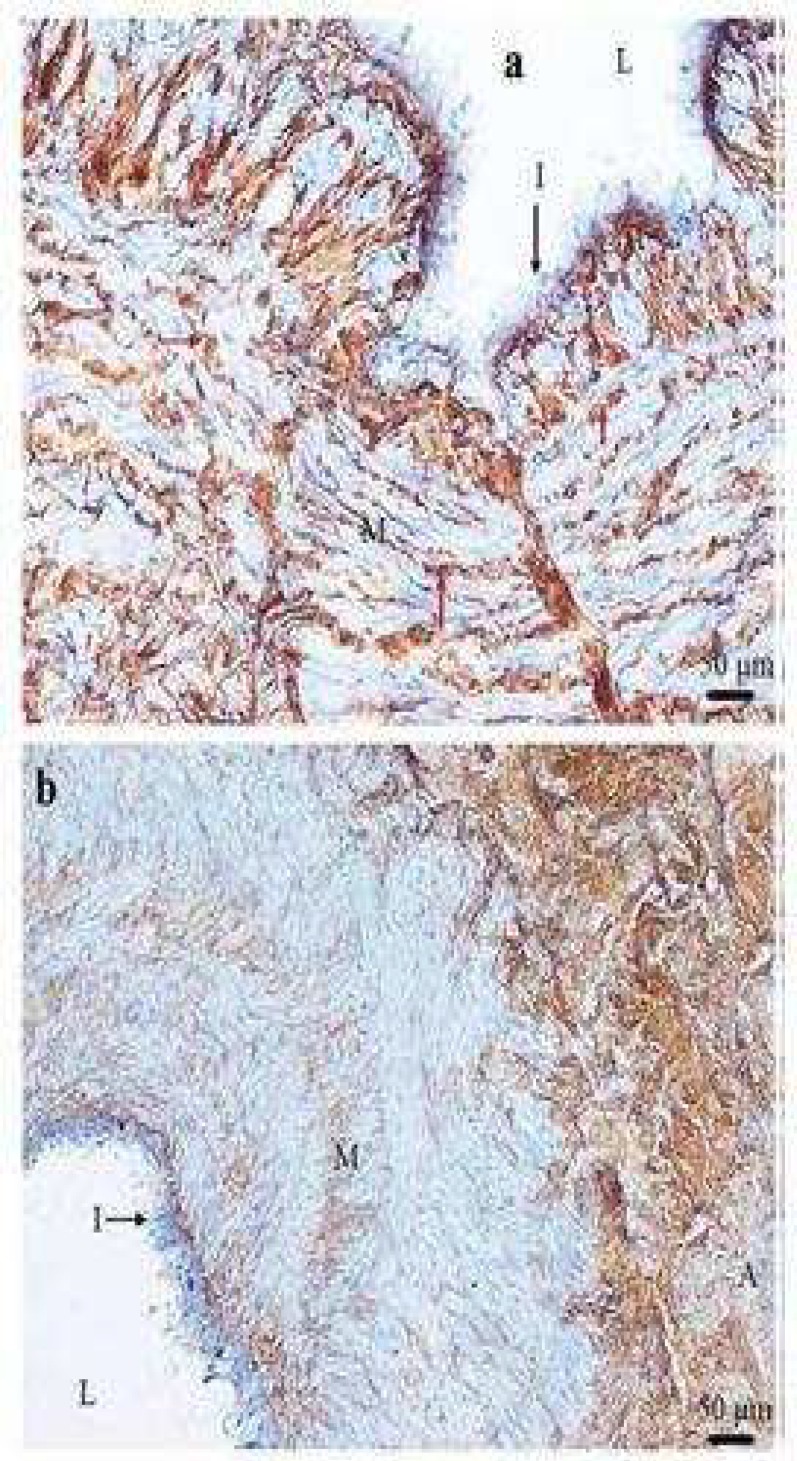
Type III collagen expression in control saphenous vein (a) and varicose vein (b). Typical transverse section of control saphenous vein (a) and varicose veins (b) stained with type III collagen antibody showing strong and weak positive staining (brown) respectively. Strong positive staining was seen in the media (M) and scattered between smooth muscle cells (red arrows). The adventitia (A) shows strong positive staining of type III collagen. I: intima; L: lumen of the vein (×200) (×100

Distribution of type III collagen in 3 (15%) varicose vein specimens showed weak staining in the media. In all control saphenous vein, the immunostaining of type III collagen was scattered between smooth muscle cells beneath the endothelial cell layer (Fig.5a).

 Immunolocalisation of type III collagen was found in the subendothelial layer of all varicose vein specimens (100%) and in the media and the adventitia of 16 specimens (85%) (Fig.5b) (Table1).

**Fig 6:**
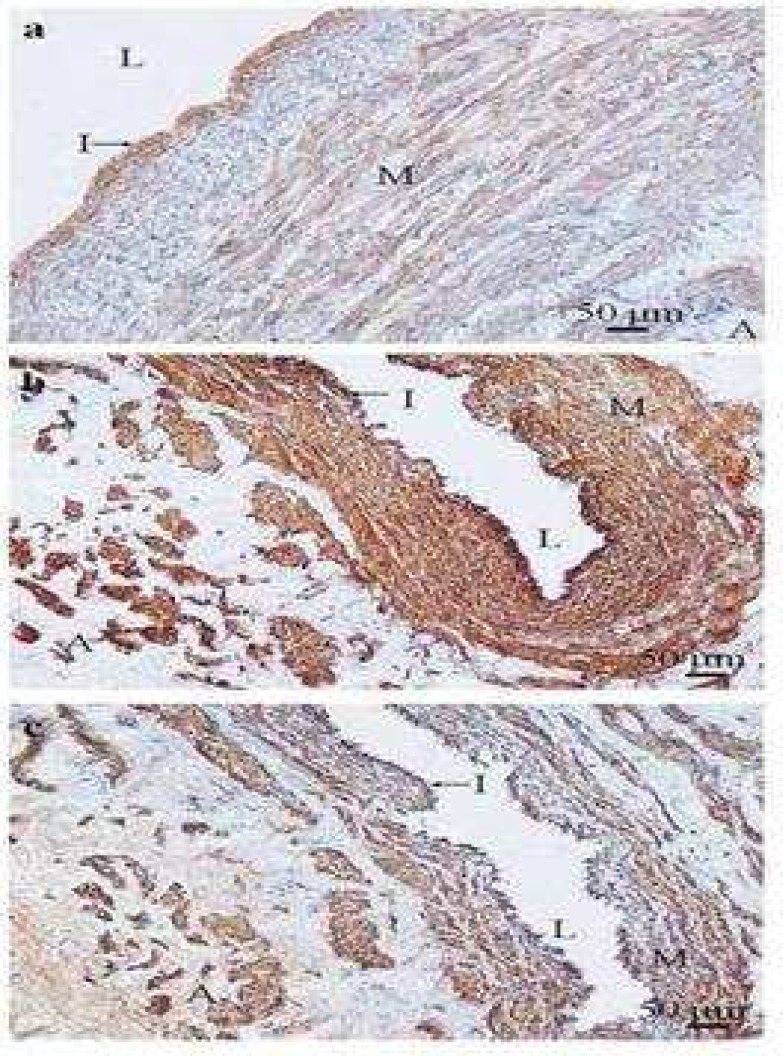
Type IV collagen expression in control saphenous vein and Type IV collagen expression compared with α- smooth muscle actin expression in varicose vein. Typical transverse section of control saphenous vein (a) stained (brown) with type IV collagen antibody showing strong positive staining in the intima (I). The media (M) shows positive staining for type IV collagen in the smooth muscle cell basement membranes. Typical transverse section of the same varicose vein stained with type IV collagen (b) and α -smooth muscle actin (c) antibodies. The positive staining (brown) of type IV collagen was found in the intima (I) which indicated the type IV collagen components of basement membrane (arrow). Type IV collagen components of basement membrane were also detected smooth muscle cell basement membranes in the media (M) and the adventitia (A). The immunostaining distribution pattern of both type IV collagen (b) and α -smooth muscle actin (c) antibodies staining indicated the role of type IV collagen as a component of basement membrane in the media and the adventitia which surrounds smooth muscle cells. L= lumen of the vein (×100

Type IV collagen staining of control (Fig.6a) and varicose saphenous (Fig.6b) veins was found in the intima and the collagenous components of SMC basement membranes in the media and the adventitia. Type IV collagen immunostaining in 16 varicose vein specimens (80%) revealed the same pattern as α -SMA staining in the media and the adventitia (Table 1).

In all the specimens of control and varicose vein (100%), strong staining of type IV collagen was detected in the intimal, medial and adventitial layers. Excepting the intimal layer which stained strongly with type IV collagen, the appearance of type IV collagen staining in the media and the adventitia of all control saphenous vein specimens was the same as α-SMA staining and demonstrates intact basement membrane around the smooth muscle cells in those areas (Table 1).

**Electron microscopy**

**Fig 7:**
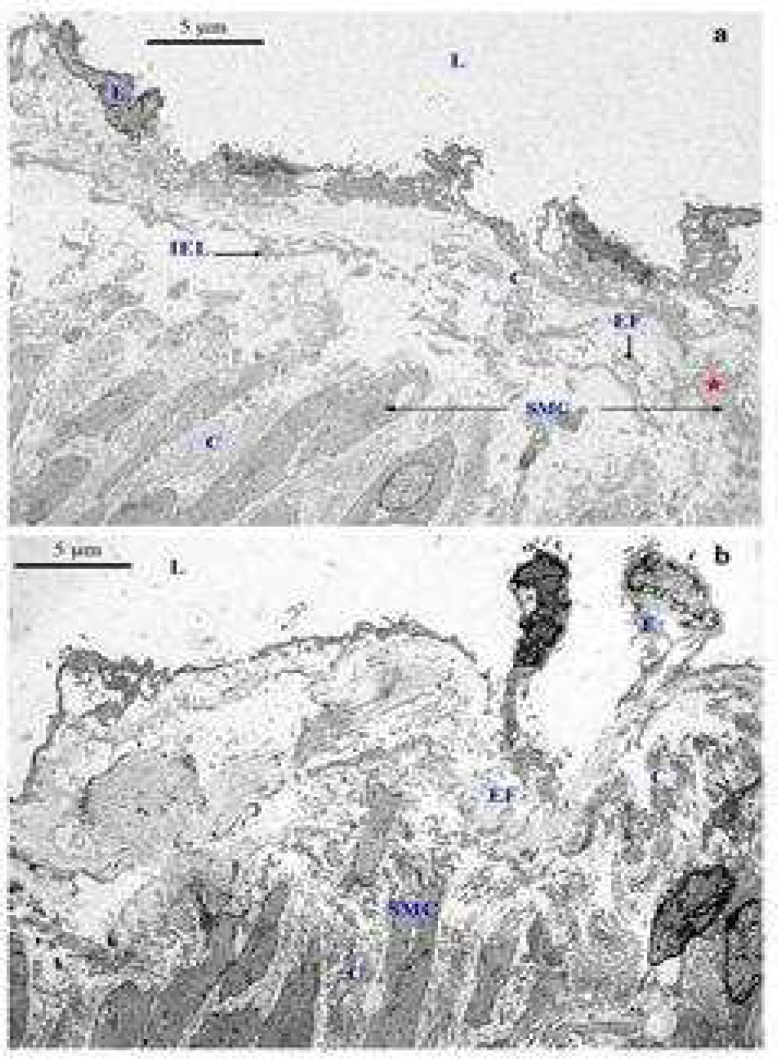
Transmission electron microscopic examination of the structure of the intima of control saphenous (a) and varicose vein (b). The structure of the intima consists of endothelial cells (E), which cover the intimal surface and collagen fibres (C), elastic fibres (EF) and few smooth muscle cells (red star). The internal elastic lamina (IEL) was fragmented in some areas and shows a loss of continuity due to ageing (a). The structure of the intima consists of endothelial cells (E), which cover the intimal surface and collagen fibres (C), elastic fibres (EF). The collagen and elastic fibres separate the intima from media as an internal lamina (b). L: lumen of the vein; SMC: smooth muscle cell (uranyl acetate-lead citrate: ×2500

**Fig 8:**
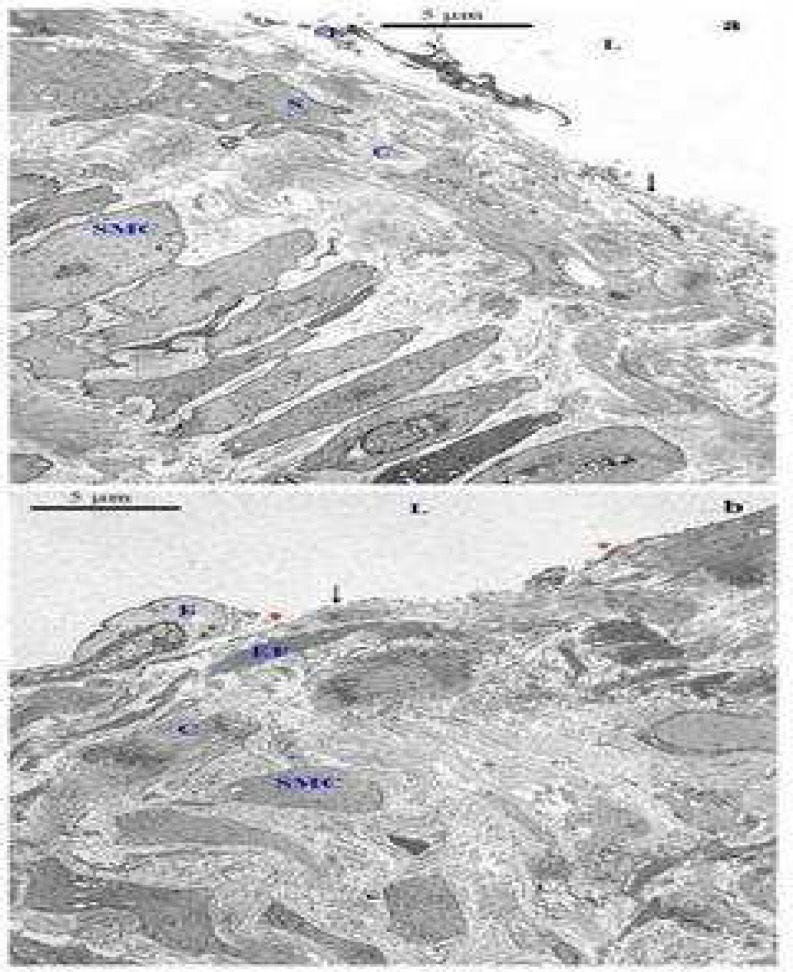
Transmission electron microscopic examination of the structure of the intima of control saphenous (a) and varicose veins (b). An endothelial cell (E) is detached from the intimal layer and increased thickness of collagen (C) is seen in this area. The arrow shows the area of missing endothelial cells. Endothelial cells (E) are detected in some parts of the intimal layer whereas in other parts they are completely missing (arrow). The elastic fibres (EF) and collagen (C) contribute to the thickening of this area. It shows more collagen accumulation in the subendothelial layer region which lost endothelial cells. The variability of endothelial cell membrane was identified tin some regions of missing endothelial cells (red arroweds). L: lumen of the vein; S: smooth muscle cell in the intimal layer; SMC: smooth muscle cell (uranyl acetate-lead citrate: ×2500

Transmission electron microscopical investigation identified changes in the varicose vein structure compared with control samples. Ultrastructural examination of 3 varicose vein specimens (15%) showed that elastic fibres were as thick as the internal elastic lamina in some areas, in which contrasts with the rest of varicose vein specimens which no internal elastic lamina was detected. In 17 out of 20 (85%) specimens of control saphenous vein (Fig.7a,b) an internal elastic lamina was detected between intima and media but it was not identified in the rest of the specimens (15%). In 18 out of 20 (90%) specimens of varicose vein, endothelial cells were detached or missing in most parts of the vein wall (Fig.8a). In three out of twenty, (15%) specimens of control saphenous vein detachment and loss of endothelial cells in most parts of the specimens was detected. In both varicose and control saphenous veins the thickness of collagen fibres in subendothelial layer appeared to be increased in regions which had lost endothelial cells, compared with (Fig.7a). However, thickness of collagen fibres in area where the endothelial cells are lost in varicose veins (Fig.8a) appeared to be greater than in the few areas of endothelial cells loss in control saphenous veins (Fig.8b).

**Fig 9:**
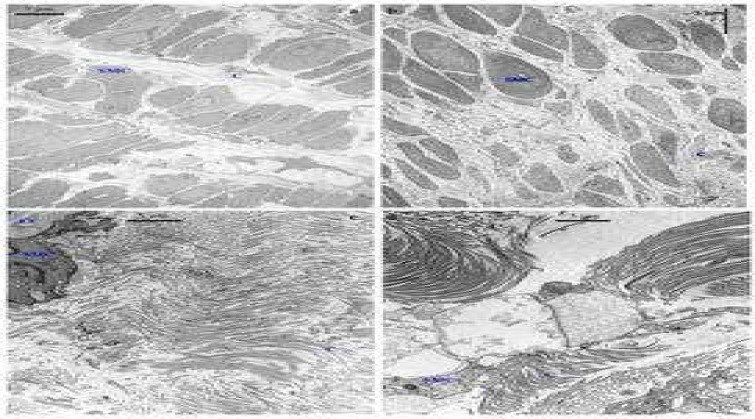
Transmission electron microscopic examination of the structure of the media of control saphenous (a, c) and varicose vein (b, d) Smooth muscle cells (SMC) in the area close to the intima are separated by variable bands of collagen (C) and are regularly arranged as a bundle. Collagen fibres are more concentrated between the bundle of smooth muscle cells compared with the space between each smooth muscle cell in the same bundle (a). Smooth muscle cells (SMC) in the area close to the intima are separated by variable bands of collagen (C) and are not arranged regularly as a bundle (b). Regularly organized collagen fibres in the media were identified. The elastic fibres (EF) surrounded (arrows) the smooth muscle cell (c). Collagen fibres (C) were irregularly organized (d). SMC: smooth muscle cell (uranyl acetate-lead citrate stain: ×10000)

 Ultrastructural examination of the tunica media in the varicose vein showed disorganization of smooth muscle cells close to the intima in 17 (85%) specimens when compared with normal organized arrangement of smooth muscle cells in 18 (90%) of control saphenous vein specimens. Smooth muscle cells in the control saphenous vein specimens were more closely arranged and showed their regular sheet-like organisation when compared with those varicose vein tissues which showed wide separation of the smooth muscle cells by an increased amount of extracellular matrix and variable bands of collagen fibres (Fig.9a,b).

The smooth muscle cells close to the adventitia in 15(75%) varicose vein specimens were not arranged closely and regularly when compared with organisation of smooth muscle cells in the same area in 18 (90%) of control saphenous vein specimens. In two specimens of control saphenous vein (10%) disarrangement of smooth muscle cells close to the adventitia was identified. An accumulation of collagen fibres filled the space between the smooth muscle cells in the media of 16 (80%) varicose vein specimens. These fibres were arranged more irregularly when compared with regularly organized collagen fibres in control saphenous veins (Fig.9c,d). In some parts of the media of 4 (20%) varicose vein specimens, elastic fibres around the smooth muscle cells did not appear to be fragmented. In the rest of the varicose vein specimens (20%) there was an organized and regular distribution of collagen fibres in the media. In 15 (75%) specimens of control saphenous vein collagen fibres were regularly organized in the media and distributed between the bundles of smooth muscle cells rather than in the space between each smooth muscle cell in the same bundle. In 5 (25%) specimens of control saphenous vein, collagen fibres were irregularly organized between the smooth muscle cells in the media. In 17 (85%) of control saphenous vein specimens elastic fibres were found around the smooth muscle cells in the majority of the media that were examined and were not fragmented. 

The adventitia in varicose and control saphenous veins consists of smooth muscle cells, *vasa vasorum *and collagen fibres. The smooth muscle cells in the adventitia of 19 (95%) varicose vein specimens were separated by collagen fibres not as closely packed as in the control saphenous vein. There were no significant differences between the varicose vein and the control specimens with respect to the pattern of endothelial cells and smooth muscle cells in *vasa vasorum*.

## Discussion

One of the strongest arguments for varicose vein wall changes is based on the changes observed in the composition of the connective tissue of the varicose vein wall. In the present study, immunostaining showed that bundles of longitudinal and circular smooth muscle cells in all of the control saphenous veins were completely separated, whereas, only in 65% of varicose vein specimens could smooth muscle bundles be recognised in the media. Moreover, among the specimens of varicose veins which show separated longitudinal and circular bundles of smooth muscle cells in the media, the thickness of each bundle appeared to be varied in different parts of the vein wall. This finding confirmed the observation of Stücker et al. that the layer size of circularmuscle in the media was reduced in parts of varicose veins (5).

Although some investigators have confirmed disorganization of smooth muscle cells in the media (27,28), others have also detected disorganization of muscle bundles in control saphenous veins of subjects with advancing age (29).

Ultrastructural examination of the tunica media in the present study confirmed disorganization of smooth muscle cells close to the intima and the adventitia in most varicose vein specimens, while well organized smooth muscle cells were identified in the equivalent regions of most control saphenous vein specimens. These ultrastructural findings are in concordance with studies of other researchers who have reported disorganization or irregular arrangement of smooth muscle cells in the media of varicose veins compared with control saphenous veins (6,22). Moreover, in the media of control saphenous vein specimens, smooth muscle cells in each bundle were more closely arranged compared with varicose veins which were clearly separated. This seemed to be due to the increase in the muscle bulk plus the intervening connective tissue, coupled with the wide separation of the smooth muscle cell bundles. On TEM examination, collagen fibres were arranged more irregularly in most varicose vein specimens when compared with regularly organized collagen fibres in most control saphenous veins. In addition, fragmentation of the medial elastic fibres was identified in most of varicose vein specimens compared with control saphenous veins. Some studies have reported an increase in amounts of smooth muscle cells or their activity in varicose veins (30,31), whereas, others found reduced amounts of smooth muscle cells due to replacement by connective tissue (6,22).

Immunostaining of the adventitia in this study confirmed previous observations (28) that this layer in varicose veins did not exhibit a distinct difference from control saphenous vein. However both immunostaining and the TEM of *vasa vasorum *in the vein wall showed no difference between varicose veins and control saphenous veins in the present investigation. This result cannot agree with the report of Badier-Commander et al. which showed that the vessels of the *vasa* *vasorum *had a larger and more irregular diameter and appeared to exhibit increased wall thickness in varicose veins (32). The emphasis in understanding how vessel wall remodeling occurs is valuable because it can explain some of the venous wall changes in varicose vein development. Collagens, as the most abundant ECM component, are the matrix proteins best associated with matrix remodeling in angiogenesis or hypertension (33).

Among the various vascular wall collagens, types I, III, and IV play critical roles in extracellular remodeling during angiogenesis. It has been showed that types I, III, and IV collagen are accumulated in vascular wall in patients and in animal models of hypertension (34), but there were no analysis of collagen type III in previous studies. These immunohistological and TEM results showed alterations in the distribution pattern of type I, III, and IV collagen in the wall of varicose veins compared with control saphenous veins. The immunohistochemical pattern of type I collagen in our results revealed strong staining in the subendothelial region of most varicose vein specimens as did control saphenous vein specimens which is an indication of an increase of this type of collagen in the subendothelial region. 10% of varicose vein specimens showed weak staining of type I collagen in the subendothelial region and the adventitia of the vein wall but no staining in the media.

The subendothelial region of all varicose and control saphenous vein specimens showed strong type III collagen staining. Type III collagen was seen in the media of some varicose vein specimens (15%) but the staining was weak. Our findings which showed strong staining of type III collagen in both the subendothelial and medial layers compared with type I collagen in varicose veins, do not quantify the level of collagens. Other investigators have quantified the overproduction of type I collagen and decreased production of type III collagen in varicose veins compared with control saphenous veins (20,21) (35-37). However, their observations were based on collagen fibres derived from cultures of varicose vein smooth muscle cells do not explain the distribution of collagen types in the three layers of veins. Investigation of the distribution of type IV collagen is an important factor in neointima formation in human saphenous veins, the early steps of endothelial morphogenesis (38). The distribution of type IV collagen in the intima of both varicose and control saphenous veins revealed intact basement membrane in most parts of all specimens which may identify an absence of the angiogenesis processes. The immunostaining pattern of distribution of type IV collagen was compared to the pattern of distribution of α - SMA in both varicose and control saphenous veins to detect any SMC basement membrane degradation. There were no differences between these patterns in most varicose vein specimens. Although the increase of type IV collagen was identified in the non-dilated portions of varicose veins compared with control saphenous veins, there is no evidence of alteration of type IV collagen distribution in varicose veins when compared with control saphenous veins (9).

Our results using both light and electron microscopy studies confirm significant alterations of morphology of the media of varicose veins when compared with control saphenous veins. In addition, collagen fibres were distributed more irregularly in most varicose vein specimens when compared with the regularly organized collagen fibres in the majority of control saphenous veins. These alterations in the distribution of pattern of type collagen to the media suggest that the pathological abnormality in varicose veins may be due to the loss of muscle tone as result of the breakup of its regular structure by the collagen fibres. These findings only described some changes in terms of distribution of these types of collagen in the intima and media of varicose vein walls compared with controls which may result in venous wall dysfunction in varicosis.
